# OD-MVSNet: Omni-dimensional dynamic multi-view stereo network

**DOI:** 10.1371/journal.pone.0309029

**Published:** 2024-08-15

**Authors:** Ke Pan, Kefeng Li, Guangyuan Zhang, Zhenfang Zhu, Peng Wang, Zhenfei Wang, Chen Fu, Guangchen Li, Yuxuan Ding

**Affiliations:** 1 College of Information Science and Electrical Engineering, Shandong Jiaotong University, Jinan, Shandong, China; 2 Shandong Zhengyuan Yeda Environmental Technology Co., Ltd., Jinan, Shandong, China; Purdue University, UNITED STATES OF AMERICA

## Abstract

Multi-view stereo based on learning is a critical task in three-dimensional reconstruction, enabling the effective inference of depth maps and the reconstruction of fine-grained scene geometry. However, the results obtained by current popular 3D reconstruction methods are not precise, and achieving high-accuracy scene reconstruction remains challenging due to the pervasive impact of feature extraction and the poor correlation between cost and volume. In addressing these issues, we propose a cascade deep residual inference network to enhance the efficiency and accuracy of multi-view stereo depth estimation. This approach builds a cost-volume pyramid from coarse to fine, generating a lightweight, compact network to improve reconstruction results. Specifically, we introduce the omni-dimensional dynamic atrous spatial pyramid pooling (OSPP), a multiscale feature extraction module capable of generating dense feature maps with multiscale contextual information. The feature maps encoded by the OSPP module can generate dense point clouds without consuming significant memory. Furthermore, to alleviate the issue of feature mismatch in cost volume regularization, we propose a normalization-based 3D attention module. The 3D attention module aggregates crucial information within the cost volume across the dimensions of channel, spatial, and depth. Through extensive experiments on benchmark datasets, notably DTU, we found that the OD-MVSNet model outperforms the baseline model by approximately 1.4% in accuracy loss, 0.9% in completeness loss, and 1.2% in overall loss, demonstrating the effectiveness of our module.

## Introduction

The goal of Multi-View Stereo (MVS) reconstruction is to utilize a collection of images taken from different angles to reconstruct a three-dimensional model representation of a scene. This method is widely applied in 3D reconstruction [1] and finds numerous applications in areas such as augmented reality, autonomous driving, smart cities, and cultural heritage preservation. MVS is an image-based passive 3D reconstruction technique that offers advantages such as high reconstruction accuracy, wide field of view, low cost, and ease of scalability when compared to active 3D reconstruction methods using tools like laser scanners and depth cameras [2].

Traditional MVS techniques [3–7] optimize depth values by leveraging the projection relationships between multiple camera views, achieving success in ideal Lambertian scenes. However, these techniques face difficulties in dealing with dense matching on reflective surfaces and enhancing the reconstruction integrity of scenes with weak textures. Additionally, external variables like lighting intensity and viewing angles significantly impact reconstruction results. To overcome these limitations, depth learning-based MVS algorithms have been proposed.

In contrast to traditional MVS methods, recent learning-based approaches have improved reconstruction for most MVS datasets. By harnessing the powerful feature extraction capabilities of convolutional neural networks, these networks can extract stronger features from overlapping images. Subsequently, these networks build a three-dimensional cost volume, derive a depth map for each view, and use depth map fusion methods to recover the three-dimensional surface of the scene.

The MVS methods based on deep learning can be divided into two types: voxel-based MVS and depth map-based MVS. The voxel-based approach utilizes trained networks to regress the occupancy of each voxel, but such methods incur significant memory consumption [8, 9], but voxel representation requires a large amount of memory. Another reconstruction approach involves estimating the depth for each view and then regressing and merging the depth maps to create the final 3D point cloud model.

Depth map-based methods use depth maps as an intermediate layer, enabling the generation of more accurate three-dimensional models [10–13]. Deep learning-based MVS methods encode and extract features from both local and global information in the scene, greatly enhancing the robustness of multi-view stereo feature matching. They can consider factors such as specularities, reflections, and changes in environmental lighting, benefiting the reconstruction of low-texture and non-Lambertian surface regions, thereby significantly improving the completeness and overall quality of reconstruction.

Although deep learning methods have made some progress in enhancing the quality of 3D reconstruction, there is still room for further improvement in the completeness of feature extraction and the accuracy of depth map estimation. This indicates that deep learning-based 3D reconstruction techniques are still in their early stages. Firstly, these networks have convolutional kernels of fixed size with limited receptive fields. Therefore, features extracted from these networks may lead to a decrease in the quality of matching costs, especially when there is a significant difference in the camera poses between the source and reference images. Secondly, the memory consumption of the network increases cubically with the increase in scene resolution. Therefore, high memory requirements limit the applicability of reconstruction strategies based on MVSNet to high-resolution images, despite their excellent performance on low-resolution images. Thus, it can be seen that existing deep learning-based MVS methods encounter difficulties in reconstructing high-resolution images.

To address these issues, we propose a dynamic feature extraction module that can adapt to different image scales. Without compromising spatial resolution, this module leverages feature maps to extract dense long-range information. In the regularization aspect, we also design a 3D channel attention module. This module, through adaptive learning of channel attention weights, enhances the accuracy of depth estimation, thereby improving the overall effectiveness of 3D reconstruction.

The contributions we make are as follows:

1. We introduce the omni-dimensional dynamic atrous spatial pyramid pooling (OSPP) module in parallel configuration for feature extraction. By extracting long-range information and capturing multi-scale contextual information, OSPP improves the density of inferring the depth map.
2. To enhance the regularization capability of the cost volume, we suggest the use of a 3D attention module. Consequently, by effectively aggregating important information in the cost volume, we can address and improve the issue of feature mismatch.

## Related work

### Neural Radiance Fields-based View Synthesis

Traditional view synthesis methods typically utilize techniques such as stitching multiple views together to achieve the desired effect. However, such methods impose many constraints on the target scene, thereby affecting the generalization performance of the related approaches. With the development of differentiable neural rendering techniques, Mildenhall et al. [14] proposed Neural Radiance Fields (NeRF), which uses Multi-Layer Perceptrons (MLPs) to approximate the mapping relationship between the captured viewpoint and various parameters of the 3D scene, and then generates the final view through volume rendering techniques. However, NeRF faces issues such as slow training speed, low generalization performance, and the inability to synthesize visually pleasing multi-scale views. Consequently, a significant amount of research based on this work has explored improvements in network performance and rendering effects.

In terms of improving training efficiency, Müller et al. [15] proposed a method of hashing encoding different-scale input views and extracting view features as network inputs, which enhances training efficiency and rendering effects, reducing training time costs while adding extra storage space. KiloNeRF [16] utilizes multiple small MLP network structures [17], with each MLP serving as an implicit representation of a portion of the scene model, thereby accelerating rendering speed and reducing training costs. However, such methods are only suitable for scenes at fixed scales, where the distance from the camera to the target object is approximately constant. When dealing with complex multi-scale scenes, NeRF’s method of sampling only one ray per pixel leads to aliasing problems, resulting in excessive blurring and artifacts in the rendered results. Employing a strategy of sampling multiple rays per pixel would reduce rendering efficiency.

SSDNeRF [18] uses a diffusion model to learn a generalizable prior of neural radiance fields from multi-view images of different objects. Ling et al. [19] introduced a large-scale scene dataset called DL3DV-10K, which provides comprehensive benchmarks for novel view synthesis and supports learning-based 3D representation techniques to obtain large-scale universal priors.

### Structure from motion

Structure from Motion (SFM) is a three-dimensional reconstruction technique that recovers the structural information of a scene based on a sequence of two-dimensional multi-view images. In the reconstruction process, cameras capture the target scene through freely taken photos along non-fixed paths. Features corresponding to the spatial points are extracted and matched from the sequence of photos, establishing the geometric relationship between the target scene and the cameras. This information is then used to gradually reconstruct the three-dimensional information of the target scene.

The algorithm mainly consists of geometric-based traditional methods and deep learning-based approaches. Geometric-based traditional methods optimize camera poses primarily by minimizing reprojection errors or photometric errors between images [20]. Deep learning-based approaches are mainly divided into predicting image depth information and camera poses. In SFM-Learner [21], networks for learning image depth information and learning camera poses are proposed, and depth information and camera poses are combined into the loss function to generate supervisory signals. Chen et al. [22] introduced optical flow into this framework to reduce the impact of dynamic regions on camera pose estimation. Since the photometric loss on optical flow decreases much more easily than the photometric loss on depth information and camera poses, the backward gradients are mainly determined by optical flow. Gordon et al. [23] replaced optical flow with residual flow to enhance depth and camera pose learning. Although these methods effectively improve reconstruction quality, they fail to recover the absolute scale of depth and camera poses.

Wang et al. [24] designed a depth unsupervised SFM network specifically for facial reconstruction, exploring facial features and using multi-view geometric constraints to train accurate face poses and depth maps. Chen et al. [25] proposed an end-to-end model with a highly correlated volumetric multi-view motion structure, achieving more precise feature matching and reconstruction by incorporating a 4D correlated volume.

### Multi-view stereo reconstruction

The three-dimensional reconstruction methods can be broadly categorized into traditional MVS reconstruction methods and MVS methods based on deep learning.

#### Traditional MVS reconstruction

In early research, Furukawa et al. [3] designed a multi-view stereo vision algorithm characterized by its accuracy, density, and robustness. By employing strategies such as stereo matching, grid expansion, and redundancy elimination, this algorithm effectively improved the precision of 3D reconstruction models. Subsequently, Galliani et al. [26] introduced the Gipuma method in 2016, based on the PatchMatch algorithm. Gipuma uses a checkerboard propagation strategy, dividing image pixels into red and black colors, and enhances reconstruction efficiency through neighborhood propagation mechanisms. Gipuma is a large-scale parallel method for multi-view 3D reconstruction that not only enhances depth consistency across multiple views but also significantly improves reconstruction speed, thereby boosting the efficiency of large-scale scene 3D reconstruction networks.

However, a common issue among these traditional multi-view 3D reconstruction methods is their unreliable handling of weakly textured regions with inconsistent photometry, resulting in surplus noise and incomplete recovery, making precise 3D model reconstruction challenging.

The open-source methods such as Colmap and OpenMVG are representative approaches in classical MVS reconstruction. They achieve the representation of dense scenes through the principles of view consistency and optical consistency.

Colmap, proposed by Schonberger et al. [5] in 2016, is an open-source 3D reconstruction system. It uses NCC (Normalized Cross-Correlation) as a metric for optical consistency between images, employs Patch-based match for depth transfer [27], and utilizes the GEM algorithm for additional depth map optimization. Building upon incremental 3D reconstruction algorithms, Ullman introduced a novel SFM technique that can further jointly infer pixel-level depth and normal [28]. Due to the inevitable redundancy in images, for dense scenes, Colmap encounters some issues with depth map integrity and continuity. The inferred depth map contains numerous gaps, and the system operates too slowly.

OpenMVG, proposed by Moulon et al. [18], is an open-source library for 3D reconstruction. This open-source software encapsulates a rich set of reconstruction algorithms and has significant applications in photogrammetry, computer vision, and robotics. While OpenMVG can accurately compute multi-view geometric poses and its model algorithms are stable, certain modules within the open-source library suffer from nested complexity, low flexibility, and a lack of large-scale SFM processing algorithms.

#### Learning-based MVS

With the continuous development of deep learning, researchers are utilizing deep learning techniques to enhance 3D reconstruction algorithms. Compared to traditional methods, multi-view 3D reconstruction based on deep learning generates more accurate and complete point clouds. These methods utilize deep learning technology to perform dense correspondence calculations and recover 3D points.

Early studies only applied deep learning to a single step of the reconstruction algorithm rather than developing end-to-end learning methods. In 2015, Han et al. [29] proposed a block matching system called MatchNet, which consists of a deep convolutional network to extract features from blocks and a network composed of three fully connected layers to calculate the similarity of the extracted features. In 2016, Choy et al. [30] introduced a new recurrent neural architecture for multi-view 3D reconstruction. This network can obtain image information of one or more object instances from any viewpoint and output a complete mesh model of the instance object. Moreover, it does not require any image annotations or object class labels for training and testing. While GCNet [31] employed a three-dimensional convolutional neural network (3D-CNN) for the regularization of cost volumes. These methods addressed certain issues but were relatively complex to implement and had limitations.

MVSNet [10] constructs a cost volume from the feature maps extracted from the images and employs 3D-CNN regularization and regression of the cost volume (which involves a large number of model parameters) to accomplish depth estimation. Additionally, the coarse depth map generated from the fixed scale of feature maps and cost volume also affects the reconstruction result. Yao et al. [11] proposed RMVSNet, which reduces the number of model parameters by using GRU instead of 3D-CNN. However, compared to MVSNet, RMVSNet has lower accuracy and requires longer training time. MVSCRF [32] is also presented as a solution to the MVSNet problem, utilizing Conditional Random Fields (CRFs) to constrain the smoothness of depth mapping, thus achieving regularization of the cost volume. Cascade-MVSNet [12], to generate high-resolution depth maps in a coarse-to-fine manner, adopts standard feature pyramid encoding geometric methods to construct the cost volume and regresses depth maps at different scales. Similar to Point-MVSNet [33], CVP-MVSNet [13] constructs the cost pyramid in a coarse-to-fine manner, significantly reducing the model’s parameter count. Fast-MVSNet [34] focuses on improving reconstruction efficiency. This network encodes dependencies between local pixels through a small-scale neural network and further optimizes depth mapping using a Gaussian-Newton layer.

### Attention mechanism

As is well known, human perception is greatly influenced by attention [35]. An important aspect of the human visual system is that people do not attempt to process the entire scene all at once. Instead, individuals employ a series of local glimpses and selectively focus on salient parts to better capture visual structures [36]. Some recent endeavors [37, 38] have integrated attention mechanisms to enhance the performance of CNNs in large-scale classification tasks.

Wang et al. proposed a residual attention network using an encoder-decoder attention module [37]. The augmented feature mapping enables the network to resist noisy inputs and perform well. We separate the learning processes of channel attention and spatial attention rather than directly computing a three-dimensional attention map. The independent attention generation process significantly reduces computational costs and parameter overhead, making it suitable as a plug-and-play module for existing foundational CNN architectures.

The attention mechanism is a commonly used technique in computer vision and natural language processing, which mimics the cognitive process of humans. Hu et al. [38] introduced a compact Squeeze-and-Excitation (SE) module to exploit relationships between channels, aiming to improve the performance of convolutional networks without increasing network complexity. It first employs a squeezing operation (also known as global pooling) to extract global statistical information for each channel feature map. Then, it uses an excitation operation to adaptively adjust the weights of each channel to enhance the feature expression capability in the network. Integrating the SE module into existing network structures is straightforward. However, previous methods overlooked the importance of maintaining spatial and channel features for enhancing cross-dimensional interactions. Therefore, to achieve fine allocation and processing of spatial information, CBAM [39] added a spatial attention module on top of the SE block.

While these attention mechanisms effectively focus on spatial and channel information, they do not take into account depth information in the three-dimensional cost volume. Since the values in the three-dimensional cost volume reflect the similarity between features, different depths at the same channel and spatial position may have similar confidences due to feature similarity. Traditional attention mechanisms only consider channels and spatial positions, failing to adequately address the more crucial depth information in the cost volume. To better incorporate attention to depth information in the cost volume, we propose a depth attention mechanism.

## Methodology

We employ an iterative optimization approach to gradually increase the size and accuracy of the inferred depth map, aiming to reduce memory and time consumption. In the scenario of having one reference image and N source images, the model utilizes a coarse-to-fine strategy to progressively regress a fine-grained depth map with the same resolution as the reference image.

**Fig 1:**
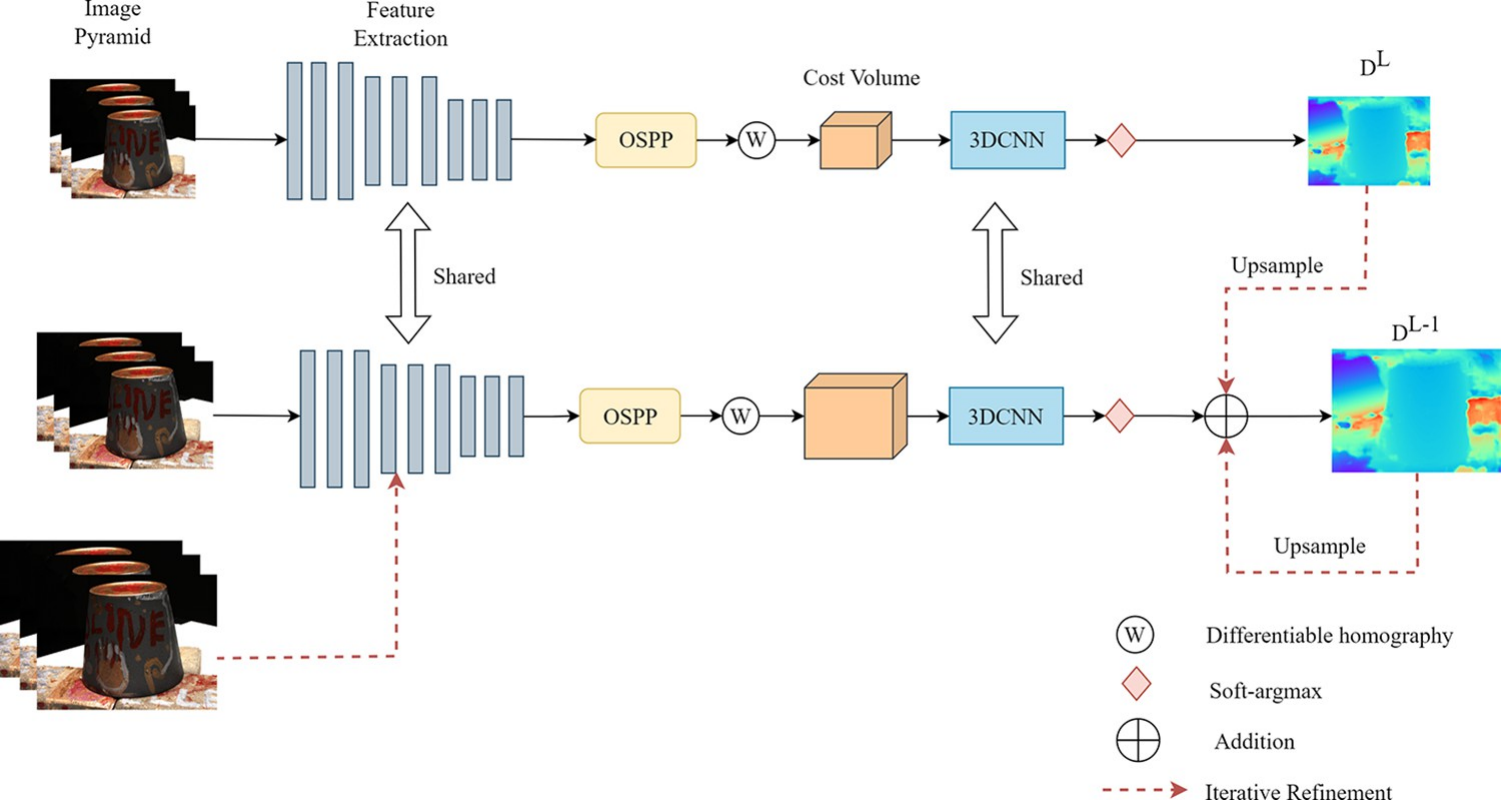
The architecture of OD-MVSNet.

We accomplish this task through four subprocesses: feature extraction, cost volume regularization, upsampling on the depth map, and depth map optimization. The proposed OD-MVSNet architecture is illustrated in Fig 1. Firstly, the reference image and source images are downsampled to form an image pyramid. The feature extraction module processes all input images to obtain image features at different scales. Next, a feature volume is constructed through homography deformation, and a cost volume corresponding to the resolution of the coarsest image is built using variance calculation. The cost volume is then regularized to generate a probability volume, and the depth map D_L_ is predicted using soft-argmax. Subsequently, local cost volumes are iteratively constructed, and depth residuals are estimated to obtain the depth map D_0_ at the original resolution. Finally, depth maps of all images are filtered and fused to obtain a dense point cloud of the scene.

### Omni-dimensional dynamic atrous spatial pyramid pooling

**Fig 2:**
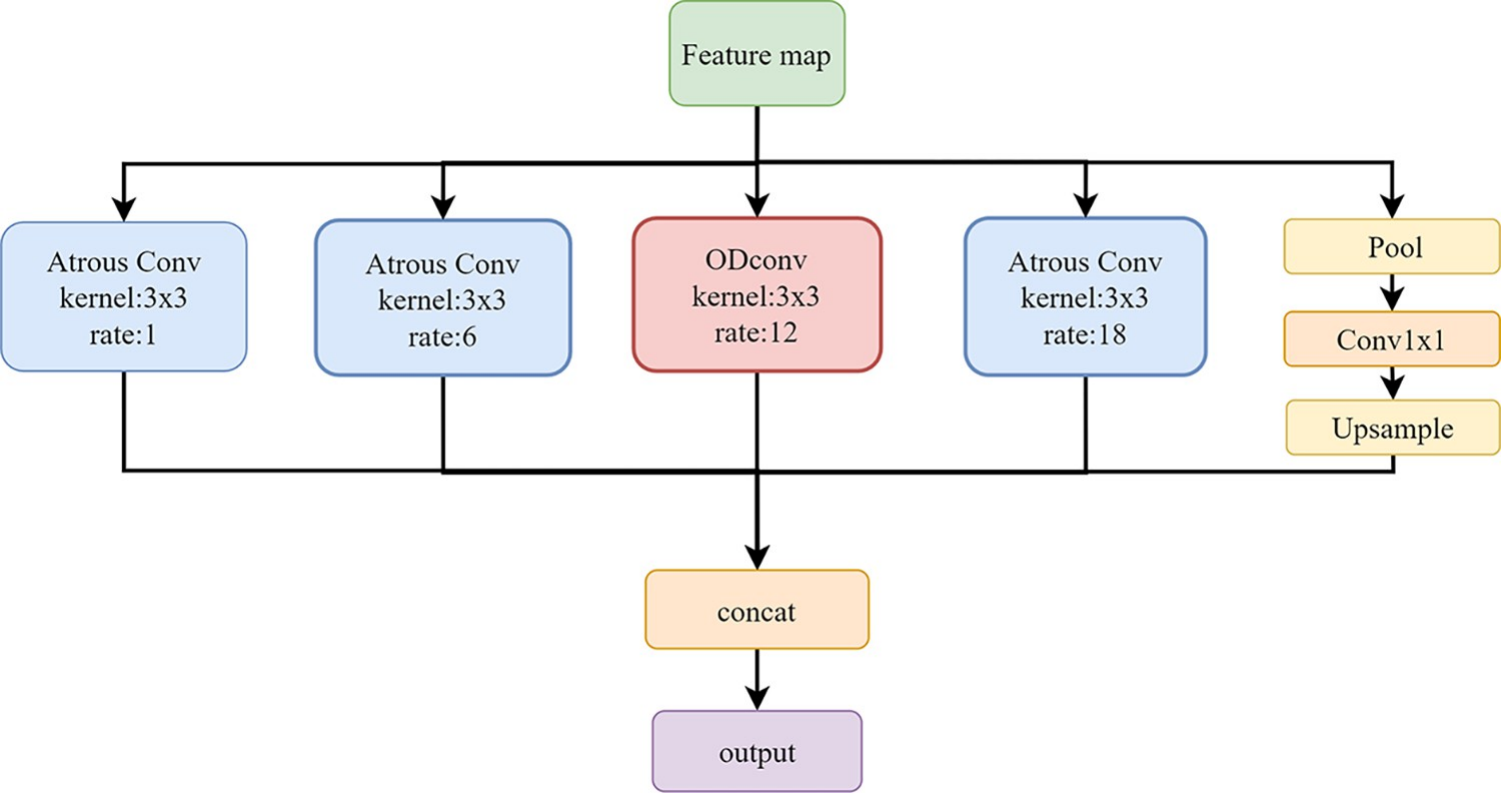
The omni-dimensional dynamic atrous spatial pyramid pooling module.

When the convolution operation is applied repeatedly to the feature map, the spatial resolution decreases. To address this issue, we have introduced the OSPP, which is a parallel convolution module that utilizes multiple dilation rates to gather multi-scale contextual information. As illustrated in Fig 2, OSPP consists of atrous convolution and dynamic convolution.

**Fig 3:**
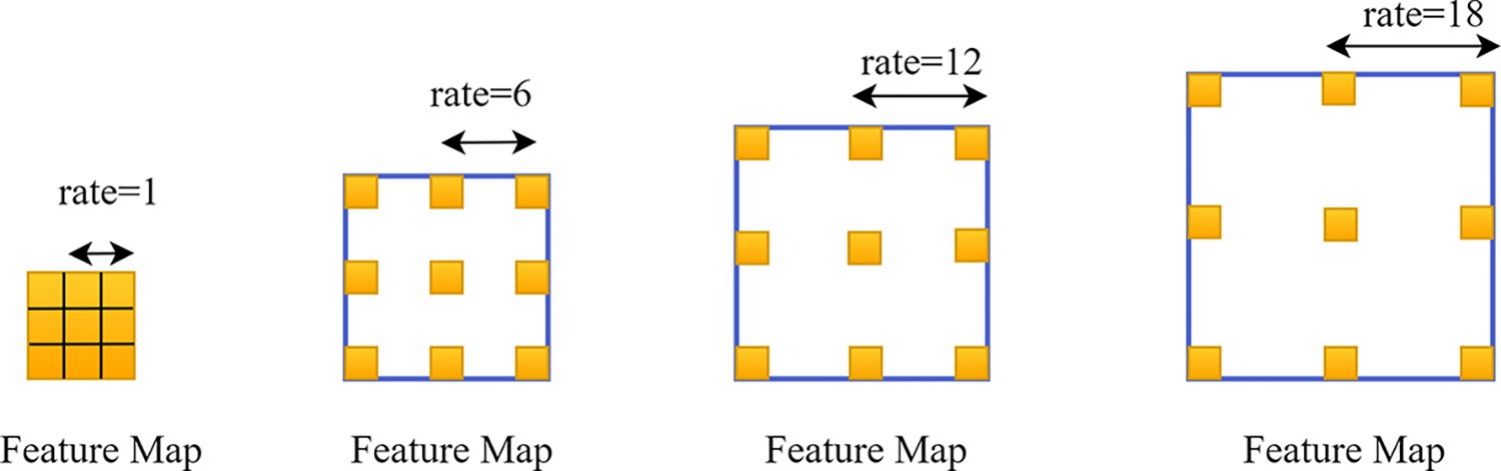
The atrous convolution.

The use of atrous convolution helps maintain spatial resolution. Setting the dilation rate in dilated convolutions is a critical factor as it determines the receptive field size of the convolutional kernel on the input image. As illustrated in Fig 3, adjusting the dilation rate allows for obtaining receptive fields of different scales, thereby capturing multi-scale contextual information. Features extracted with different dilation rates are further processed and fused in different branches to obtain the final output.

The convolution operation on the mth pixel of the feature map x using a filter w with h parameters is given by Eq (1).


$$y(m)=\underset{h}{\sum }x[m+r.h]w[h]$$ (1)

Where the variable y(m) represents the feature map obtained through convolutional operations, where r is the stride used for sampling the input signal. To capture multi-scale object information, this module constructs convolutions with different dilation rates to create feature maps with varying receptive fields. The feature maps undergo two types of convolution operations: 1x1 and 3x3 convolutions. If the dilation rate is too high or the spatial resolution of the feature map is low, atrous convolutions may fail to encode global contextual information, potentially leading to a degradation similar to that observed in 1x1 convolutions. To prevent such degradation, global pooling is employed to extract image features.

**Fig 4:**
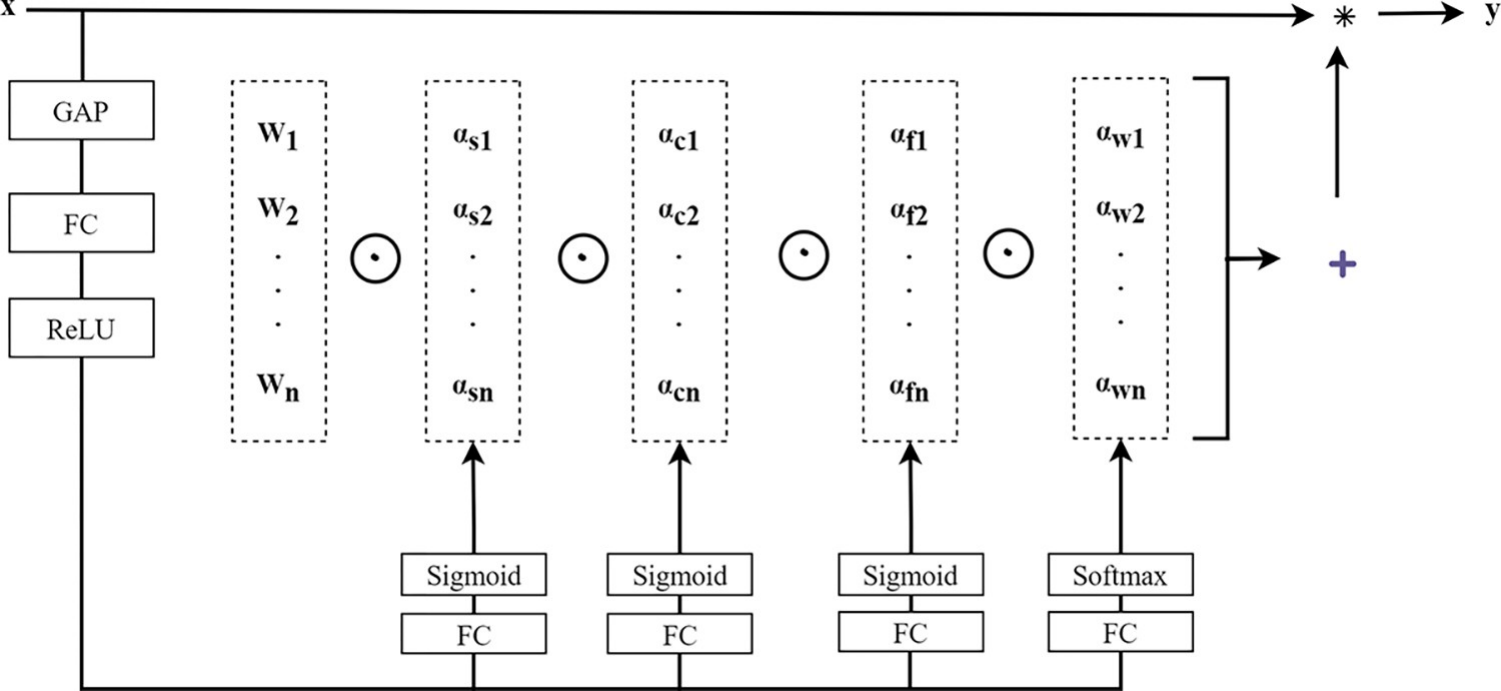
The omni-dimensional dynamic convolution module.

We integrate atrous convolutions with dynamic convolutions to enhance the expressive capacity of the model. The fundamental concept of dynamic convolutions involves adaptively adjusting convolutional parameters based on the input image. To learn attention within the convolutional kernel across all four dimensions of the convolutional kernel space, omni-dimensional dynamic convolution (ODConv) employs a novel multidimensional attention mechanism and parallel strategies, as illustrated in Fig 4. ODConv is capable of acquiring attention values for the entire convolutional kernel, as depicted in Eq (2), to capture rich contextual information, thereby significantly improving the feature extraction capabilities of convolution.


$$y=(\alpha_{w1}⊙\alpha_{f1}⊙\alpha_{c1}⊙\alpha_{s1}⊙W_{1}+\ldots +\alpha_{wn}⊙\alpha_{fn}⊙\alpha_{cn}⊙\alpha_{sn}⊙W_{n})*x$$ (2)

Where α_Wi_ represents the attention scalar for convolutional kernel W_i_, while α_si_, α_ci_, and α_fi_ denote three newly introduced attentions calculated along the spatial dimension, input channel dimension, and output channel dimension of convolutional kernel W_i_, respectively. The symbol ⊙ denotes element-wise multiplication along different dimensions of the kernel space.

### Normalization-based 3d attention module

Due to factors such as non-Lambertian surfaces and occlusion, the original cost volume often contains a lot of noise, leading to overfitting of the network. To further filter out noise in the cost volume and generate a probability volume for depth inference, we employ a multi-scale 3D CNN for cost volume regularization. The overall architecture of the regularization module adopts an encoder-decoder structure.

**Fig 5:**
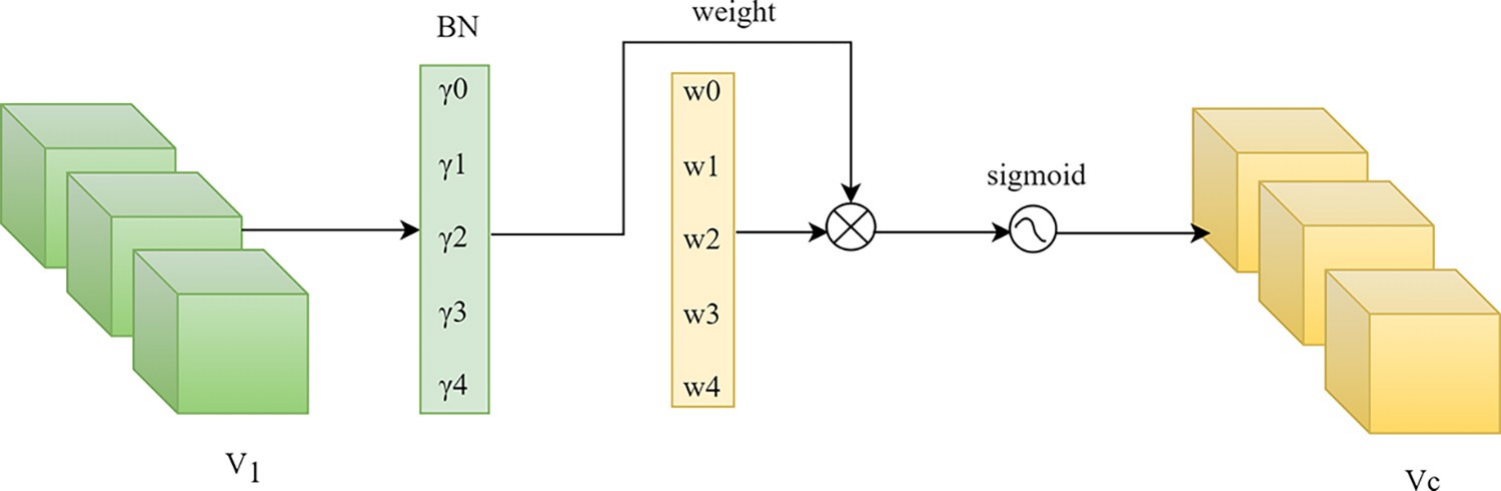
Normalization-based 3D attention module.

Since there are errors in feature matching across multiple key points in the cost volume, similar confidence scores arise for different depth points within the cost volume, resulting in inaccurate depth estimations. Recognizing the capability of attention mechanisms to focus the model on crucial information by assigning varying weights, we employ an attention-based approach to address the issue of feature mismatch. In this study, we propose an efficient and lightweight 3D attention module, referred to as normalization-based 3D attention module (N3DAM) and integrate it into the regularization of the cost volume. The structure of the regularization module is shown in Fig 5.

In the 3D attention module, we utilize the scaling factor from Batch Normalization (BN), as shown in Eq (3). The scaling factor, equivalent to the variance in BN, reflects the magnitude of changes in each channel, indicating its importance. The magnitude of the variance signifies the extent of channel variations. Channels exhibiting pronounced changes imply richer information content and higher importance, while channels with minimal changes possess singular information and lower importance.


$$B_{out}=BN(B_{in})=\gamma \frac{B_{in}-\mu_{B}}{\sqrt{\sigma_{B}^{2}+\epsilon }}+\beta$$ (3)

Where μ_β_ and σ_β_ represent the mean and standard deviation of the mini-batch B, respectively. γ and β denote trainable parameters for the affine transformation (scale and shift). γ serves as the scaling factor for each channel, and its weight is defined by Eq (4).


$$W_{\gamma }=\gamma_{i}/\underset{j=0}{\sum }\gamma_{j}$$ (4)

**Fig 6:**
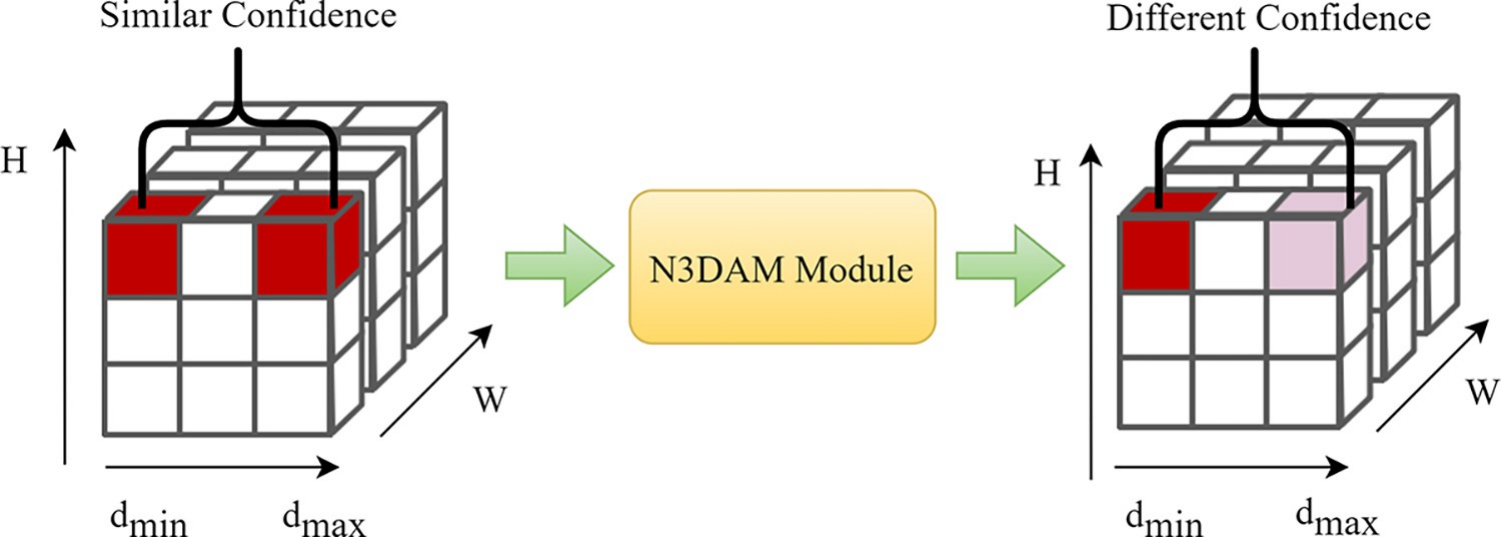
The problem of similarity confidence at different depth.

This module enhances or diminishes the similarity confidence at different depths by computing attention weights, thereby addressing this issue. The schematic diagram of this module is illustrated in Fig 6.

The calculation formula for the channel attention module is defined in Eq (5), where M_c_ represents the ultimately obtained output features.


$$M_{c}=sigmoid(W_{\gamma }(BN(V_{1})))$$ (5)

### Loss function

Similar to the existing MVSNet framework [10], we employ a supervised learning strategy that uses the L1 paradigm to measure the absolute difference between the ground truth and the estimated depth values. For each training sample, our loss is defined as in Eq (6):

$$Loss=\overset{L}{\underset{l=0}{\sum }}\underset{p\in \Omega }{\sum }\|D^{l}(p)-{D_{GT}}^{l}(p){\|}_{1}$$ (6)

Where D_GT_ represents the ground truth depth values, D denotes the depth values estimated by the model, and Ω is the set of effective pixels with ground truth measurements.

## Experiments

In this section, we demonstrate the performance of the MVS framework through a comprehensive set of experiments. Next, we first present the dataset and then analyze the results of the experiments.

### Dataset

The DTU dataset [40] is a large-scale indoor scene dataset designed for Multi-View Stereo reconstruction. It comprises a total of 27,097 training samples, each composed of 124 scenes under distinct lighting conditions. Each scene is characterized by 49 precise camera positions and references structured light scans. Each scan consists of 49 images captured under 7 different lighting conditions. The dataset also includes normal information and intrinsic and extrinsic parameters of cameras from various perspectives. To ensure a fair comparison between OD-MVSNet and other reconstruction methods, this study adopts the same training, validation, and testing sets as utilized in MVSNet.

### Implement details

#### Training

We trained and evaluated OD-MVSNet on the DTU dataset. During training, we set the input image resolution to 160×128 with N = 3 views. We constructed a two-layer image pyramid, where the first stage followed a coarse-to-fine strategy similar to CasMVSNet, with the number of depth planes K set to 48, uniformly sampled in the range of 425 mm to 921 mm. In the subsequent stage, each pixel had K = 8 depth residual hypotheses for refinement in-depth estimation. OD-MVSNet optimization was performed using the Adam optimizer over 40 epochs on a single NVIDIA RTX A5000 GPU. The initial learning rate was set to 0.001, and the batch size was set to 16, with iterative halving at the 10th, 12th, 14th, and 20th epochs.

#### Testing

We conducted evaluations of the trained models on the DTU test dataset. Similar to previous methodologies, we utilized five views and employed the same depth map fusion technique to generate point clouds. Evaluation of the DTU dataset followed Yao’s approach, where the output depth maps were transformed into predicted point clouds. Subsequently, a comparison was performed with the ground truth point clouds using the official Matlab code provided by the dataset.

#### Evaluation metrics

The evaluation metrics for the MVS algorithm can be categorized into two forms: distance measures and percentage measures. Distance measures include accuracy and completeness. Specifically, the accuracy of the reconstruction results refers to the proximity between the reconstructed points and the ground truth point cloud, quantified as the mean or median of the absolute distances between each reconstructed point and the corresponding point in the ground truth point cloud. Completeness of the reconstruction results represents the number of points in the ground truth model covered by the reconstructed model, measured as the mean or median of the absolute distances between each point in the ground truth point cloud and the corresponding point in the reconstructed point cloud. Typically, the average of accuracy and completeness is employed as an overall score to characterize the overall quality of the reconstruction results.

### Results on the DTU dataset

**Fig 7:**
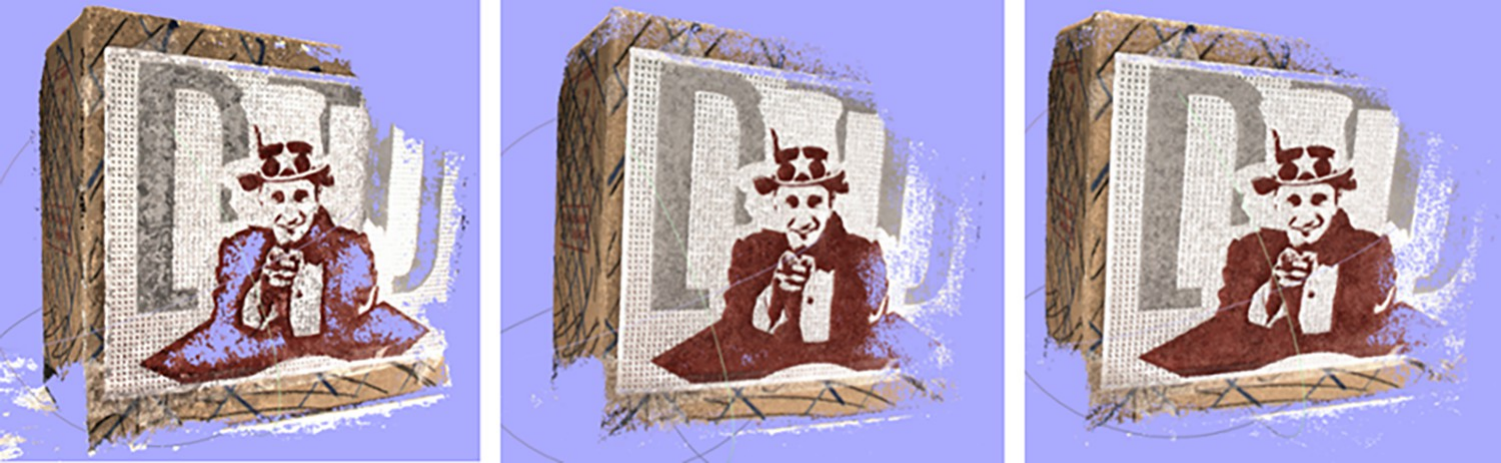
Qualitative results of scan9 of the DTU dataset. (a) MVSNet, (b) CasMVSNet, (c) Ours.

The proposed model is compared with traditional methods and state-of-the-art learning-based approaches, as outlined in Table 1. The relatively lower integrity scores indicate that the point clouds generated using this method exhibit fine details, particularly in regions characterized by weak textures and boundaries. Qualitative results for point clouds are presented in Fig 7. Our approach demonstrates superior completeness in textureless regions compared to MVSNet [10] and CasMVSNet [12]. The reconstructed point cloud model exhibits smoother surfaces and sharper boundaries, validating the efficacy of utilizing the OSPP module to enhance both density and completeness in the reconstructed point cloud.

**Table 1:** Quantitative results on the DTU dataset (lower is better).

Method	Acc. (mm)	Comp. (mm)	Overall(mm)
Furu [3]	0.613	0.941	0.777
Tola [41]	0.342	1.190	0.766
Camp [42]	0.835	0.554	0.695
Gipuma [6]	0.283	0.873	0.578
Colmap [5]	0.400	0.664	0.532
MVSNet [10]	0.396	0.527	0.462
R-MVSNet [11]	0.383	0.452	0.417
Point-MVSNet [33]	0.342	0.411	0.376
Cascade-MVSNet [12]	0.325	0.385	0.355
AA-RMVSNet [43]	0.376	0.339	0.357
Vis-MVSNet [44]	0.369	0.361	0.365
OD-MVSNet (Ours)	0.294	0.398	0.346

The comparison of GPU memory consumption and the time required for predicting depth maps on the DTU dataset is shown in Table 2. Compared to other models, our method demonstrates significantly reduced memory requirements and relatively decreased computation time.

**Table 2:** Comparison of GPU memory consumption and inference time on the DTU dataset.

Method	GPU memory(GB)	Time(s)
MVSNet [10]	15.4	1.48
Point-MVSNet [33]	12.7	3.04
HSF-MVSNet [45]	21.2	3.1
OD-MVSNet(ours)	12.1	1.35

### Ablation experiments

**Fig 8:**
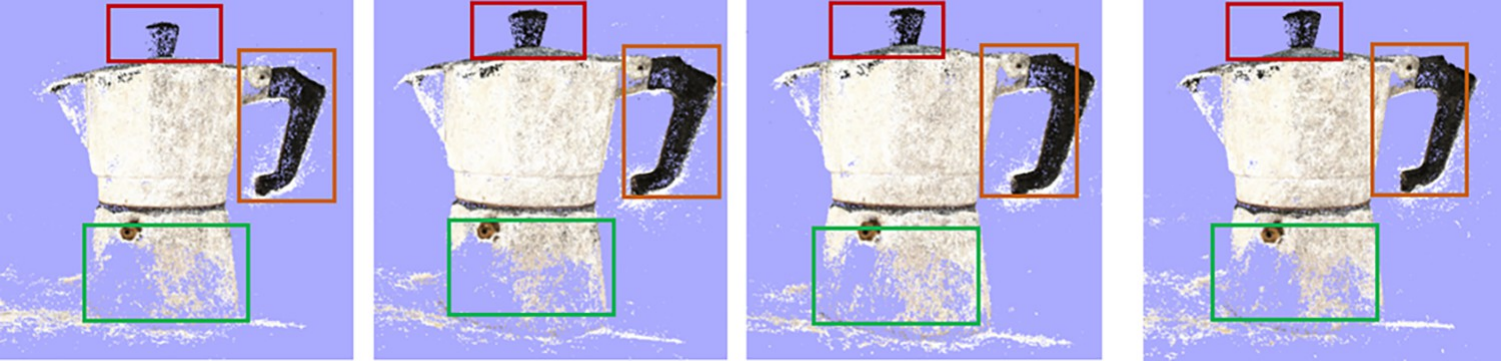
Comparison of point cloud reconstructed images from different modules. Baseline, Baseline+OSPP, Baseline+N3DAM, OD-MVSNet.

To validate the effectiveness of the proposed method, we conducted ablation experiments on the DTU dataset. Table 3. presents the evaluation results of the two proposed enhancement modules. The first row displays the metrics of the baseline model. The second row presents the evaluation metrics after incorporating the OSPP module into the original model. The third row shows the evaluation metrics for the entire OD-MVSNet. Merely adding the OSPP module resulted in a marginal decrease of 0.002 millimeters in the overall score. This indicates that, owing to the OSPP module, the network can efficiently extract features by incorporating contextual information from distant sources. Furthermore, with the inclusion of N3DAM, the model’s accuracy and completeness decreased significantly by 0.008 millimeters and 0.011 millimeters, respectively, validating the efficacy of the proposed 3D attention module. Both modules contribute to improvements in the Acc, Comp, and Overall metrics, affirming that OD-MVSNet effectively enhances the accuracy and completeness of three-dimensional reconstruction. The point cloud reconstruction effect after adding different modules is shown in Fig 8.

**Table 3:** Ablation studies on the DTU dataset.

Method	Acc. (mm)	Comp. (mm)	Overall (mm)
Baseline	0.308	0.407	0.358
Baseline+OSPP	0.306	0.405	0.356
Baseline+N3DAM	0.300	0.396	0.348
Baseline+OSPP+N3DAM	0.294	0.398	0.346

## Conclusion

Large-scale scene three-dimensional reconstruction remains challenging due to the substantial memory requirements of the task, coupled with the tendency to overlook fine details in pixel features. We propose an end-to-end network, termed OD-MVSNet, which operates in a coarse-to-fine fashion for efficient and accurate depth estimation in MVS. In comparison to other MVS methods based on cascade structures, OD-MVSNet exhibits enhanced robustness, thereby yielding more precise depth estimations even in coarse stages. This approach significantly reduces GPU memory demands and computation time. Experimental results substantiate the effectiveness and efficiency of the proposed method.

## Supporting information

S1 Table Detailed performance comparison on DTU data set. (XLSX)
